# Epigallocatechin-3 Gallate Inhibits *STAT-1/JAK2/IRF-1/HLA-DR/HLA-B* and Reduces CD8 MKG2D Lymphocytes of Alopecia Areata Patients

**DOI:** 10.3390/ijerph15122882

**Published:** 2018-12-15

**Authors:** Fatma N. Hamed, Andrew J. G. McDonagh, Sarah Almaghrabi, Youssef Bakri, Andrew G. Messenger, Rachid Tazi-Ahnini

**Affiliations:** 1 Department of Infection, Immunity and Cardiovascular disease, The Medical School, University of Sheffield, Sheffield S10 2RX, UK; Fnhamed1@sheffield.ac.uk (F.N.H.); S_maghrabi@windowslive.com (S.A.); 2 Department of Dermatology, Royal Hallamshire Hospital, Sheffield S10 2JF, UK; a.j.mcdonagh@shef.ac.uk (A.J.G.M.); a.g.messenger@shef.ac.uk (A.G.M.); 3 Laboratoire de Biologie de Pathologies Humaines Faculté des Sciences, Université Mohammed V, P.B. 1014 Rabat, Morocco; ybakri@gmail.com

**Keywords:** alopecia areata, epigallocatechin-3-gallate, STAT1, CD8+ NKG2D+ subset

## Abstract

Background: Alopecia areata (AA) is associated with Interferon- γ (IFN-γ) mediated T-lymphocyte dysfunction and increased circulating Interleukine-17 (IL-17) levels. Epigallocatechin-3-gallate (EGCG) specifically inhibits IFN-γ pathways and unlike Janus Kinase 1 and 2 (JAK1/JAK2) inhibitors (tofacitinib, ruxolitinib), EGCG is safer, more cost-effective, and is a topically active agent. Our objective is to test the mode of action of EGCG in vitro and ex vivo using HaCat, Jurkat cell lines, and peripheral blood mononuclear cells (PBMCs) of AA patients and healthy controls (HCs), respectively. Methods: distribution of T helper cells (Th1, Th17), and cytotoxic cells (CD8) in PBMCs isolated from 30 AA patients and 30 HCs was investigated by flowcytomterty. In vitro treatment of HaCat and Jurkat cells with 40 μm EGCG for 48 h was performed to measure the level of phosphorylation of signal transducer and activator of transcription protein STAT1, and replicated in ex vivo model using PBMCs of AA patients. Results: Interestingly, 40 μm EGCG is capable of completely inhibiting phosphorylation of STAT1 after 48 h in HaCat and Jurkat cells and ex vivo in PBMCs of AA patients. Based on QPCR data, the action of EGCG on p-STAT1 seems to be mediated via downregulation of the expression of JAK2 but not JAK1 leading to the inhibition of human leukocyte antigens (HLA-DR and HLA-B) expression probably via IRF-1. On the other hand, AA patients have significantly increased levels of Th1, Th17, and CD8 cells and the production of IFN-γ and IL-17 by PBMCs in AA patients was significantly higher compared to HC; *p* = 0.008 and *p* = 0.006, respectively. Total numbers of CD8+ cells were not significantly different between treated and untreated samples. However, CD8+ cells with positive Natural killer group 2 member D (NKG2D) transmembrane receptor (CD8+ NKG2D+ subset) was significantly reduced when PBMCs were treated with 20 μm EGCG for 48 h. Conclusion: These results suggest that EGCG has a synergistic action that inhibits expression of HLA-DR and HLA-B molecules via the IFN-γ pathway to maintain immune privilege in HF; also it reduces CD8+ NKG2D+ subset.

## 1. Introduction

Alopecia areata (AA) is an autoimmune disease focused on the hair follicles (HFs) and nails. It presents typically as well-demarcated areas of hair loss, which may progress to total scalp hair loss (alopecia totalis) or whole-body hair loss (alopecia universalis) in a minority of cases. Currently available treatments are limited in efficacy and frequently unsatisfactory. 

There is strong evidence of the involvement of several genes in the pathogenesis of AA including Immune related genes such as HLA class II genes (*HLA-DRA, HLA-DQA, HLA-DQB*) [1,2,3], *MICA* [2,4], *CTLA4* [2,5], *NOTCH4* [2,6], *AIRE* [7,8,9,10], *PTPN22* [11,12] *FOXP3* [2], *IL1RN* [13,14,15,16] as well as hair and skin related genes such as *MX1* [17], *ERBB3* [2,18], *PRDX5* [2,19,20] and *STX17* [2,21]. There is also evidence that stress is involved in the pathogenesis of AA via the release of corticotropin-releasing hormone, substance P, and nerve growth factor, which induce the degranulation of mast-cells and the release of inflammatory cytokines such as TNF-a, IL-1, and IL-6 [22,23,24,25]. 

The pathogenesis of AA is also thought to be a consequence of collapse of the normal physiological state of immune privilege (IP) in the HF of genetically susceptible individuals. Collapse of IP results in autoimmune attack of the originally privileged sites, leading to autoimmune diseases such as autoimmune uveitis, autoimmune orchitis, and fetal rejection. In AA, follicular IP is characterized by a lack of expression of major histocompatibility complex (MHC) classes I and II in the proximal part of the anagen HF [26,27], and the entire lower two-thirds of the anagen HF is devoid of antigen presenting cells (APCs) [28,29]. Only scant numbers of NK cells, CD4+, are found in the lower portions of the proximal hair follicle [27]. These features are thought to be mediated by immunosuppressive factors, such as TGF-β1, α-MSH, IL-10, adrenocorticotrophic hormone (ACTH), and an immunoinhibitory signal (CD200) secreted by hair follicle cells [30,31,32].

In the aberrant IP state found in AA, the cellular and molecular elements of normal HF are altered with upregulation of MHC class I and class II compared to normal control skin [32,33,34], resulting in the exposure of auto-antigens to cytotoxic T cells and subsequent infiltration of CD4+ T cells, NK, and APCs [35]. Expression of the main inducer of MHC class I, IFN-γ, which is a Th1 cytokine, is increased in the affected skin. In contrast to the normal state, IP guardians, such as TGF-β and α-MSH are downregulated in AA lesional areas [32,36,37].

**Figure 1:**
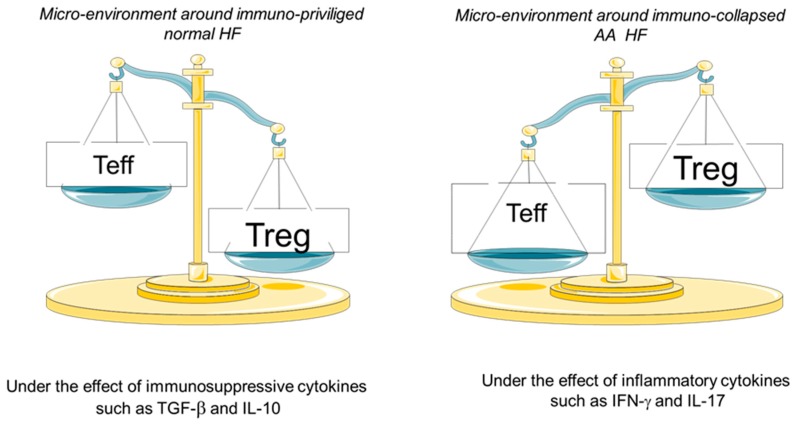
Proposed role of T cells in immune privilege (IP) collapse. In normal IP state (**left** panel), Treg and other hair follicle (HF) cells secret IP guardians such as TGF-β and IL-10, keeping the MHC class I and II expression low. As a result, the HF is devoid of inflammatory lymphocytes (Teff). In the aberrant IP state (**right** panel), the altered balance between inflammatory T cells (Th1 and Th17) and Treg is a key element in the pathogenesis. Th1 and Th17 activation results in IFN-γ secretion leading to upregulation of MHC class I and II expression.

It has been suggested that antigen identification and processing by APCs results in IFN-γ production, which in turn upregulates MHC class I with subsequent sequestration of Tc cells [38,39]. Further production of IFN-γ and upregulation of MHC class II with sequestration of Th1 and Th17 cells results in HF damage. Immune privilege collapse results in dense infiltration of T-lymphocytes of both CD4+ and CD8+ phenotypes, which is one of the histopathological hallmarks of AA [40,41]. CD4+ cells constitute 60–80% of the lymphocytic infiltrate in AA affected skin [40]. Recent investigations have addressed a number of questions such as: which CD4+ subset has the key role in AA pathogenesis and whether the effect is mediated by collapsing IP. Naïve CD4^+^ can differentiate into Th1, Th2, Th17 or CD4+ CD25+ regulatory T cells (Treg), which are characterized by their cytokine profiles [42]. For instance, the CD4+ Th1 subset secretes IFN-γ, TNF- β, and IL-2 [43]. The CD4+ Th2 subset secretes IL-4, IL-5, IL-10, and IL-13 [44]; the CD4+ Th17 subset secretes IL-17, IL-21, IL-22, IL-23, and IL-6; and Treg cells secrete TGF-β, and IL-10 [45,46]. Altering the balance between the effector and regulatory populations of CD4+ cells is a mechanism of autoimmunity in a number of diseases (Figure 1) [47].

The role of Th1 cells in AA pathogenesis is supported by the IFN-γ signature observed in lesional tissue [38,39]. Furthermore, expression of Th1 chemokines such as CXCL-9, CXCL-10, and their receptor CXCR3 is upregulated and correlated with disease activity [48]. IL-2, which is another Th1 cytokine, was found to be elevated in peripheral blood of patients with severe AA [49]. Th17 is another CD4+ cell population that has been proposed to have a role in AA pathogenesis, and IL-17 expression was found to be significantly higher in AA-affected skin compared to controls [50]. The increase in Th17 cells infiltrating lesional HFs of AA patients was associated with a reduction in FOXP3+ Treg [51,52]. Involvement of Th17 in AA might be explained by its role in reducing Treg recruitment [52] with resultant development of a pro-inflammatory micro-environment in HFs. Lew et al [53] found a single nucleotide polymorphism (rs879577) in the gene for IL-17 receptor (IL-17RA) that was significantly increased in Korean AA patients compared to healthy controls [53]. Similarly, Aytekin et al. (2014) also showed that IL-17 SNP is associated with increased susceptibility to AA [54]. Furthermore, IL-17 was significantly increased in the serum of AA patients when compared to healthy controls [55].

The NKG2D receptor, which is expressed only on the surface of activated CD8+ cells, has been previously implicated in AA by virtue of upregulation of its ligands, ULBP3 and MICA, in the dermal papilla and dermal sheath of AA HFs compared to normal [2,56]. NKG2D ligands activate the cytotoxic activity of CD8+ T cells [2,57]. Such activation is followed by IFN-γ production from cytotoxic CD8+ NKG2D+ T cells mediating inflammation and HF damage [57].

It is well known that IFN-γ mediates its action via the JAK-STAT pathway [58]. As a main inducer of IP collapse and consequently AA, it is important to understand its activation pathway. IFN-γ binds to its receptors, IFNGR1–2, followed by their dimerization and activation of Janus kinase enzymes JAK1 and JAK2. JAK enzymes are a phosphorylate signal transducer and activator of transcription protein (STAT1), which translocates into the nucleus and binds to IFN-gamma activated sequences (GAS). GAS is a specific DNA sequence response to IFN-γ binding by subsequent activation of IFN-γ dependent gene expression, which mediates the inflammatory response [59].

IFN-γ activates a large number of genes (up to 500) and the transcription factor, interferon regulatory factor (IRF-1), is among the key genes regulated by IFN-γ. IRF-1 activates a group of genes such as those involved in the transcription of antigen presenting molecules, namely MHC class I and class II, TAP [60], and adhesion molecules such as ICAM-1 and VCAM-1 [61]. It also promotes the development and function of Th1 and Tc cells [62]. IRF-1 is also considered a key negative regulator of Treg through repression of FOXP3 expression [63].

Targeting the JAK-STAT1 pathway by a chemical inhibitor has shown promising results in AA. Three JAK inhibitors have been used in AA including ruxolitinib, tofacitinib, and baricitinib. Systemic administration of ruxolitinib resulted in successful hair regrowth in nine patients in a small pilot study in 12 AA patients [58]. In a case series of 90 patients with extensive alopecia areata, 58% achieved at least 50% improvement following treatment with the JAK1/3 inhibitor tofacitinib [64]. However, tofacitinib (which is not licensed in Europe) costs ~$2000/month and can predispose to life-threatening infections. Treatment with baricitinib (a JAK1 and JAK2 inhibitor), in one patient, was reported as showing full scalp hair regrowth after nine months [65]. This drug has a high cost, side-effects, and, in this case, the patient relapsed just a few weeks after cessation of their treatment with JAK inhibitors [66]. It is, therefore, proposed to target the same pathway, but with less toxic and more specific inhibitors.

Green tea (*Camellia sinensis*), has been shown to have many health promoting effects. Catechins constitute about 40% of the dry weight of green tea and epigallocatechin-3-gallate (EGCG) is the major component accounting for 60% of the total catechin [67]. Epigallocatechin-3-gallate is a polyphenolic flavonoid (C_22_H_18_O_11_ of average molecular mass 458 Da) with anti-inflammatory, anti-oxidant, and anti-tumor properties. It has been found to have an inhibitory effect on IFN-γ signaling via the JAK-STAT pathway. It reduces STAT1 translocation into the nucleus by inhibiting phosphorylation. Downregulation of JAK1 and JAK2 enzymes has been achieved by EGCG in human oral cancer cell lines [68], and a reproducible STAT1 inhibitory effect of EGCG was shown in a study on colorectal cell lines [69]. Epigallocatechin-3-gallate has also been shown to inhibit T cell proliferation by inhibiting IL-2 [70] and to be therapeutically beneficial in experimental autoimmune encephalomyelitis [71]. In a study by Wu and colleagues [72], EGCG at a physiologically achievable concentration of 2.5–10 μm, inhibited proliferation in primary T cells isolated from C57BL mice spleen. The same experiment in human subjects was performed by Katiyar [73], applying EGCG cream topically (3 mg EGCG on 2.5 cm^2^) to normal volunteers’ skin. The EGCG was applied 30 min before UVB exposure and an inhibitory effect of EGCG on UVB induced leukocyte infiltration (neutrophil, monocytes, and macrophage) was observed [74]. No adverse effects have been recorded in human healthy volunteers after oral administration of 800 mg daily of EGCG for four weeks, which is equivalent to the EGCG content of 6–18 cups of tea daily, giving EGCG a good safety profile [74]. Additionally, EGCG is applicable in topical preparations with a good skin penetration index, to minimize possible side-effects [75].

## 2. Material and Methods

### 2.1. Blood Samples

Our study was reviewed and approved by the Institutional Review Boards and Ethics Committees at the University of Sheffield (LREC Reference Number 002651) and Sheffield Teaching Hospitals (NHS Permission Reference Number STH18941). Twenty patients with active hair loss and an established diagnosis of AA were recruited and provide consent at the Department of Dermatology, Royal Hallamshire Hospital, Sheffield, UK (details of patients are given in the Supplementary Materials, Table S4). Patients diagnosed with other autoimmune diseases or receiving immunosuppressive drugs were excluded from the study. The cases recruited included 9 with patchy AA, 5 with alopecia totalis, and 6 with alopecia universalis. Patients and healthy controls were age-matched and were all females of Caucasian ethnicity.

### 2.2. Cell Lines

HaCat (human keratinocyte line) cells were kindly provided by Professor Sheila McNeil, Dept of Materials Science and Engineering, and maintained in high glucose Dulbecco’s Modified Eagle’s Medium DMEM (Lonza) at 5–100% confluence. To harvest the cells, they were incubated in phosphate buffered saline (PBS) with 0.02% Ethylenediaminetetraacetic acid (EDTA) for 10 min, followed by a further incubation with 0.05% trypsin/0.02% EDTA (1:1) solution for 3–5 min. A Jurkat cell line was kindly provided by Vanessa Singleton, Dept of Infection and Immunity, and maintained in Roswell Park Memorial Institute medium RPMI 1640 (Lonza) at 2–10 × 10^5^ cell density. Media of both cell lines were supplemented with 10% fetal bovine serum (FBS, Gibco-BRL) and the cells maintained at 37 °C in a 5% CO_2_ atmosphere.

### 2.3. Treatment with EGCG

Cells were seeded at a density of 5 × 10^5^ per well in a 6-well plate (HaCat) or 2 × 10^5^ per mL in a T25 flask (Jurkat). After overnight incubation, they were stimulated with 50 or 100 u/mL recombinant human IFN-γ (300-02, Peprotech). After 48 h incubation, the cells were treated with 20 or 40 μm EGCG for 24 or 48 h before being harvested for RNA or protein assays. The EGCG concentrations were determined after we performed toxicity assays (Figures S1–S3 in Supplementary Materials). The EGCG (E4143, Sigma) was dissolved in water at 10 mM stock solution. Cells were incubated with IFN-γ for 48 h prior to EGCG treatment to induce STAT1 phosphorylation. The doses were 100 IU/mL IFN-γ in Jurkat or primary T cells or 50 IU/mL in HaCat cells.

### 2.4. Peripheral Blood Mononuclear Cell Separation and FACS Analysis

Peripheral blood mononuclear cells (PBMCs) were isolated from heparinized venous blood by density gradient purification using Lymphoprep as described by the manufacturer (07801, Stem Cell). They were then stained with two panels of antibodies: IL-17 or Treg panel (Tables S1 and S2). Briefly, 10^6^ PBMCs were incubated for 30 min at room temperature with blue fixable live/dead dye, washed once with PBS, and stained with antibodies targeting surface markers in each panel. The cells were then fixed and permeabilized by fix/perm buffer (transcription buffer set, 562725, BD) for 40–50 min at 4 °C, followed by intracellular staining where cells were incubated for 40–50 min at 4 °C with antibodies specific for FOXP3, IL-10 or IL-17. The cells were finally fixed in 2% PFA, staining visualized by LSR II (Becton Dickinson, Heidelberg, Germany), and further gating performed by Flow Jo software to determine frequency of T cell subpopulations. Gating of the positive population for each marker was performed based on a florescence minus one control (FMO). Unstained, single-cell controls and compensation controls were also used to set-up the experiment. All antibodies were used at dilution 1:100.

### 2.5. Western Blotting

Peripheral blood mononuclear cells or cell line lysates were prepared by homogenization in Radioimmunoprecipitation assay (RIPA) buffer (150 mM sodium chloride, 50mM Tris-HCl, pH 7.4, 2 mM ethylenediaminetetraacetic acid, 1% Triton X-100, 0.5% sodium deoxycholate, 0.1% sodium dodecylsulfate) containing a protease inhibitor cocktail (P8340-5ML, Sigma). Protein concentration was determined by the bicinchoninic acid (BCA) assay (Pierce™ BCA Protein Assay Kit, 23225, Thermofisher, Loughborough, UK). The cell lysate was boiled for 5 min in 1 × sodium dodecyl sulfate (SDS) sample buffer (B31010, Lifetechnologies, Loughborough, UK) and proteins separated by SDS-PAGE (pH8.8, 10% [37:1] acrylamide, 0.375M Tris-Cl and 0.1% SDS, Loughborough, UK). After electrophoresis, the gel was transferred to a Polyvinylidene difluoride (PVDF) membrane using the iblot system (Invitrogen, Paisley, UK). Primary antibodies used were 1:1000 monoclonal rabbit anti p-STAT1 IgG (9167, CST), 1:1000 rabbit polyclonal anti-HLA-DR IgG (ab175085), and 1:10,000 monoclonal rabbit anti-GAPDH IgG (ab128915, Abcam). The secondary antibody used was 1:10,000 polyclonal goat anti-rabbit IgG conjugated with peroxidase (4050-05, Southern Biotech, Birmingham, USA). The membrane was developed using Enhanced chemiluminescence reagent (EZ-ECL) (20-500-500, Biological Industries, Cromwell, USA) and visualized on a ChemiDoc XRS+ System (Bio-Rad).

### 2.6. Enzyme-Linked Immunosorbent Assay (ELISA)

Peripheral blood mononuclear cells were isolated from heparinized blood by density gradient purification over Lymphoprep and stimulated with 5 ng/Ml Phorbol 12-myristate 13-acetate (PMA) (P-8139, Sigma, Welwyn Garden City, UK ) and 0.1 ug/mL ionomycin (I-0634, Sigma, Welwyn Garden City, UK) for 3.5 h or left unstimulated. Supernatants were assayed for IL-17 or IFN-γ levels by IL-17 ELISA kit (KAC1591, Invitrogen, Paisley, UK) and IFN-γ ELISA kit (KHC4021, Invitrogen, Paisley, UK) according to the manufacturer’s instructions. As the unstimulated samples showed undetectable levels of protein, readings included in the analysis were from PMA/ionomycin stimulated samples.

### 2.7. RNA Extraction and cDNA Synthesis

Total RNA was extracted using TRIzol reagent method. Briefly, cells were lysed by TRIzol, and the aqueous phase was separated and collected. The RNA was then precipitated by addition of 0.5 mL of isopropanol per 1 mL of TRIzol, washed with 75% ethanol and finally re-suspended in 20 μL RNase free water.

### 2.8. Q-PCR Analysis of Gene Expression

The relative expression of genes of interest was measured by q-PCR using power SYBER green fluorescence (Life Technologies) and specific primers for the target gene (details are given in Tables S2 and S3).

### 2.9. Statistical Analysis

The Q-PCR data was exported in a Microsoft Excel file and means were calculated for the triplicate repeats in each experiment. Differential expression was determined by the ΔΔCt method where means and SD were determined for experimental repeats. An unpaired *t*-test was used to determine any significant change in gene expression using GraphPad Prism 6 software (manufacturer, city and country). For flow cytometry data, the percentage of each T-lymphocyte sub-population was compared between patients and healthy controls using a two-tailed independent *t*-test and the corrected *t*-test was used whenever the homogeneity of variance was violated. The analysis was done using SPSS version 22 (SPSS Inc., Chicago, IL, USA). Descriptive statistics are presented as the mean ± standard deviation. Graphs shown were drawn by GraphPad Prism 6 software (Prism 6, San Diego, CA, USA).

## 3. Results

### 3.1. Inhibition of IFN-γ Signalling Pathway by EGCG

**Figure 2:**
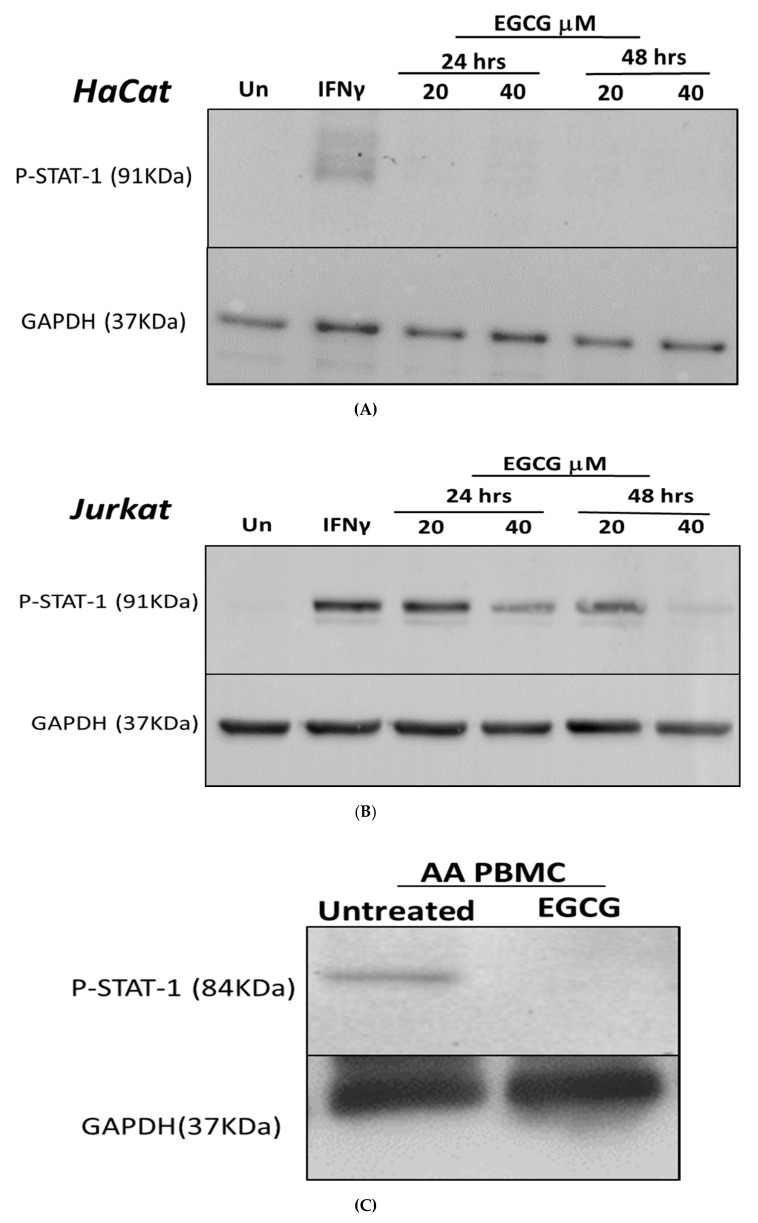
The effect of Epigallocatechin-3-gallate (EGCG) on P-STAT-1 protein in HaCat (**A**) and Jurkat cells (**B**). STAT-1 phosphorylation was induced by treating the cells with IFN-γ for 48 h. The cells were then treated with 20 and 40 µm EGCG for 24 or 48 h, with the protein levels determined by Western blotting. (**A**) HaCat cells showed marked inhibition at STAT-1 phosphorylation when treated with 40 µm for 48 h. (**B**) Jurkat cells respond to EGCG treatment in a dose-dependent manner where 40 µm dosage showed a more marked reduction in p-STAT-1 protein compared to 20 µm. A representative immunoblot is shown. (**C**) Peripheral blood mononuclear cells of alopecia areata (AA) patients were treated with 40 µm EGCG or left untreated. pSTAT1 was shown to be expressed in patients’ samples and declined after treatment with EGCG. *n* = 3, *p* < 0.05 was significant.

To investigate its effect on the IFN-γ signaling pathway (JAK-STAT), keratinocyte HaCat cells were first incubated with IFN-γ to induce STAT-1 phosphorylation, then treated with EGCG at 20 or 40 μm for 24 or 48 h. The pSTAT1 was significantly inhibited by EGCG with 20 μm and after only 24 h of treatment with EGCG (Figure 2A). There was a reduction in pSTAT1 by 35% in HaCat cells treated with 20 μm. The increase of EGCG concentration to 40 μm decreased the expression of pSTAT1 by 81% (Figure 2A). To confirm the findings in HaCat cells, we performed the same inhibition tests in lymphocyte Jurkat cells. There was a reduction in pSTAT1 of 16% in Jurkat treated with 20 μm (Figure 2B). The increase of EGCG concentration to 40 μm decreased the expression of pSTAT1 by 53% in Jurkat cells (Figure 2B). To confirm the findings in these cell lines, PBMCs from patients with AA were treated with 40 μm EGCG for 48 h after which protein was extracted and analyzed by Western blot. The pSTAT1 protein was shown to be expressed in patients’ PBMCs, and treatment with EGCG decreased pSTAT1 protein expression by 80% (Figure 2C).

### 3.2. Effect of EGCG on IFN-γ Downstream Genes

#### 3.2.1. JAK1/JAK2/STAT1 and IRF-1

**Figure 3:**
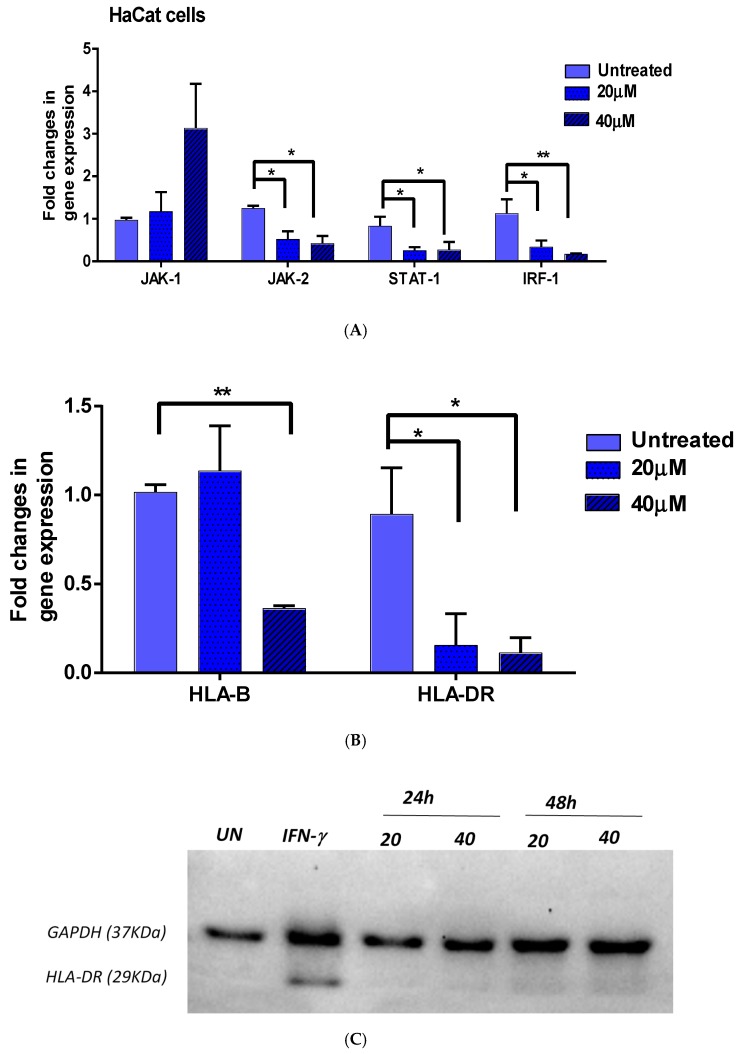
The effect of EGCG treatment on IFN-γ downstream genes in HaCat cells. The effect of EGCG treatment on IFN-γ downstream genes in the HaCat cell line. Following induction with IFN-γ, HaCat cells were treated with 40 µM EGCG for 48 h and expression of IFN-γ downstream genes was investigated by Q-PCR. CT values were normalised to GAPDH and differential expression (2^−∆∆CT) of AA candidate genes in EGCG treated samples was calculated against untreated samples (**A**) QPCR results for STAT1, JAK1, JAK2, and IRF-1. (**B**) QPCR results for *HLA-B* and *HLA-DR*. Data represented as mean ± SEM (*n* = 4). Significant difference * *p* < 0.05. ** *p* < 0.01. (**C**) Cells were induced with IFN-γ for 48 h then treated with 20 or 40 µM EGCG for 24 or 48 h. Proteins were extracted and Western blot performed using GAPDH or HLA-DR antibody.

To confirm that EGCG involved the inhibition of IFN-γ pathway we measured the expression of JAK1 and JAK2 as well as the key regulated by IFN-γ; interferon regulatory factor (IRF-1). Keratinocytes (HaCat) cells were first induced by IFN-γ, as described earlier, then treated with 40 μm EGCG for 48 h or left untreated as a control. As expected, EGCG inhibited the expression of STAT1 in keratinocytes. There was also significant inhibition of JAK2 expression in a dose-dependent manner, but JAK1 expression was not affected (Figure 3A). Expression of STAT1 downstream genes such as *IRF-1*, *HLA-DR* and *HLA-B*, was also analyzed in cultured keratinocytes. As shown in Figure 3A, the expression of *IRF-1* was significantly reduced. The reduction of *IRF-1* expression was dose dependent, suggesting that the inhibitory effect of EGCG on STAT1 did affect the expression of the downstream gene, *IRF-1* as well.

#### 3.2.2. *HLA-DR* and *HLA-B*

Because of the importance of HLA class I and class II in the immune privilege in the hair follicles and being at the end of the chain in the JAK1/STAT1 pathway, we wanted to check whether EGCG also affects the expression of these molecules at protein level. Keratinocyte (HaCat) cells were first induced by IFN-γ as described earlier, then treated with 20 or 40 μm EGCG for 24 or 48 h or left untreated as a control. The expression levels of HLA-B did not change after 48 h of treatment of the cells with 20 μm EGCG. However, a significant (*p* < 0.01) decrease in the expression of *HLA-B* was observed with 40 μm of EGCG, suggesting that only the higher dose of EGCG could have an effect on *HLA-B* expression (Figure 3B). In contrast, a significant reduction in *HLA-DR* expression was observed with the lower dose of EGCG (20 μm), which persisted with the higher dose (40 μm) (Figure 3B). To confirm the Q-PCR data, total proteins were extracted from these cells and 20 µg of protein from treated and untreated cells were loaded on SDS gel, transferred onto a membrane and hybridized with the internal control Glyceraldehyde 3-phosphate dehydrogenase (GAPDH) or HLA-DR antibody. The band corresponding to GAPDH showed that equal amounts of protein were loaded from each treatment. Induction with IFN-γ enhanced the expression of HLA-DR in HaCat cells. However, treatment with EGCG for 24 h or 48 h reduced the amount of HLA-DR proteins in the cells by more than 36% (Figure 3C). Hybridization with HLA-B antibody showed no signal, even after induction with IFN-γ, due to the low expression level of HLA-B in HaCat cells (data not shown).

### 3.3. Inflammatory Cells Th1, NKG2D+ and Th17 and Their Cytokines in AA Patients

**Figure 4:**
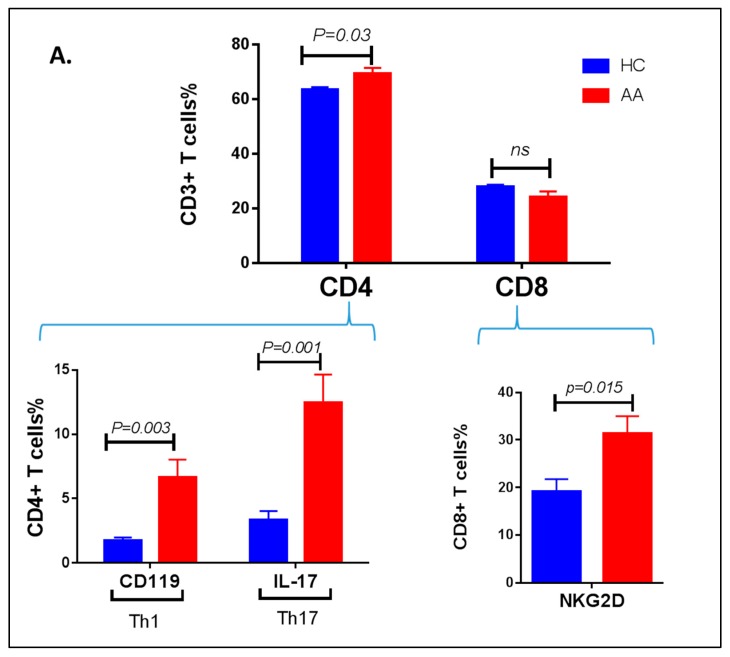

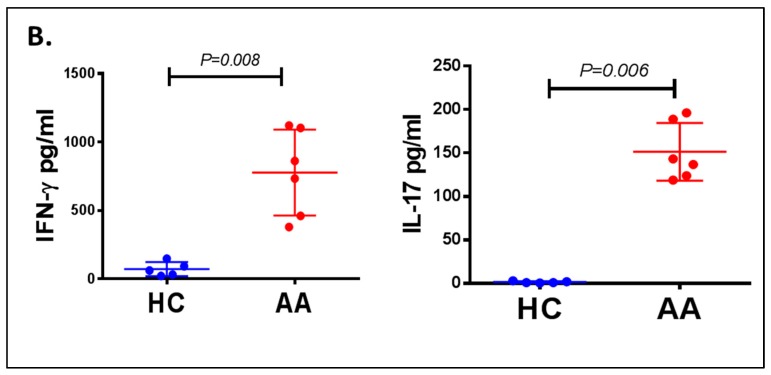
Inflammatory T cell subsets in AA. (**A**) Frequency of CD4+ and CD8+ T cell subsets of CD3+ lymphocytes and their subsets were investigated by flow-cytomteric analysis of PMBCs isolated from AA patients or HC. The CD4+ T cell pool and its inflammatory subsets (Th1 and Th17) were increased in AA compared to HC. The NKG2D+ subset of CD8+ T cells were also increased in AA. (**B**) The production of IFN-γ and IL-17 cytokines by PBMC in AA patients is significantly higher than HC upon activation with ionomycin/PMA.

To dissect the role of effector/inflammatory T cells in AA pathogenesis, a multi-color flow cytometry panel was designed to look at all possible T cell subsets that might have a potential role in the pathogenesis. Peripheral blood mononuclear cells isolated from heparinized blood of AA patients or HC were analyzed by flow-cytometry after staining the cells with CD4, CD119, and IL-17 as well as CD8 and NKG2D in the same tube. Not surprisingly, the CD4+ T cell pool was found to be higher in patients compared to HC (*p* = 0.03). Very interestingly, further dissection of CD4 T cell subsets provided an explanation of the increased CD4 T cell population in patients. Th1 as presented by IFN-γ receptor CD119 (*p* = 0.003), as well as Th17, investigated by its distinct cytokine secretion of IL-17 (*p* = 0.001), were significantly higher in AA compared to HC (Figure 4A).

The NKG2D subset of CD8 has previously been shown to have a key role in AA. Therefore, it was important to validate this in our setting. CD8+ T cell frequencies showed no difference between AA and HC, but interestingly, the NKG2D subset of CD8+ T-cells was significantly increased in AA compared to HC (*p* = 0.015). The increase in Th17 population was confirmed by an ELISA assay, which showed a significant increase in the production of the intracellular cytokines and IL-17 (*p* = 0.006) (Figure 4B). IFN-γ production was also higher (*p* = 0.008), and this can be linked to Th1 or CD8+ cytotoxic T cells.

### 3.4. The Effect of EGCG on CD8 NKG2D Lymphocytes

**Figure 5:**
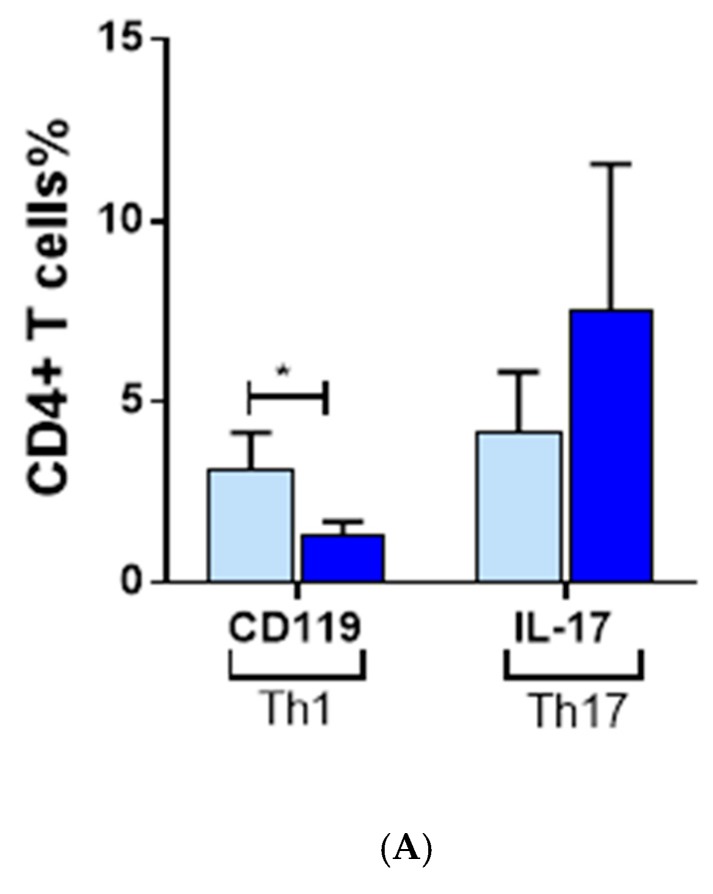

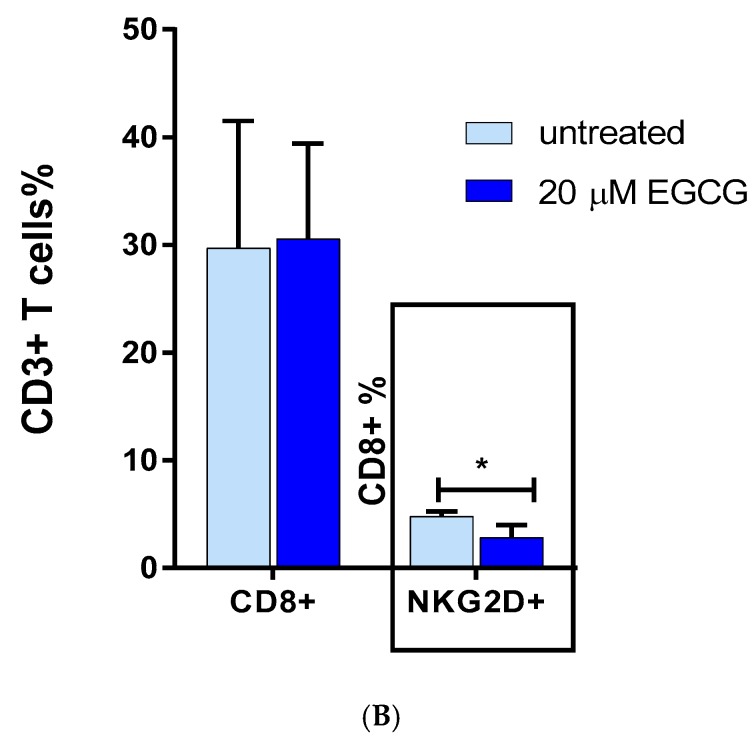
Treatment of AA PBMC with EGCG. (**A**) Distribution of Th1 and Th17 subsets of CD4+ populations after AA PBMCs were either treated with 20 µM EGCG or untreated and analyzed by flow cytometry using CD119 and Th17 antibodies respectively. (**B**) Distribution of CD8+ NKG2D+ populations after AA PBMC were either treated with 20 µM EGCG or untreated and analyzed by flow cytometry using NKG2D antibody. Each experiment was repeated three times. * *p* < 0.05.

There was a significant reduction of Th1 in treated PBMCs compared to untreated PBMCs (Figure 5A). There was also a trend showing a reduction in the number of Th17 cells in treated samples compared to untreated, but the difference was not significant (Figure 5A). However, there was no significant difference of Th2 between treated and untreated AA samples. Similarly, total numbers of CD8+ cells were not significantly different between treated and untreated samples. However, CD8+ NKG2D+ subset was significantly reduced when PBMC were treated with 20 μm EGCG for 48 h (Figure 5B). It should be mentioned here that the number of CD25+ FOXP3+ Treg cells did not change significantly when cells were treated with 20 μm EGCG (data not shown).

### 3.5. The Effect of EGCG on Pro- and Anti-Inflammatory Cytokines

**Figure 6:**
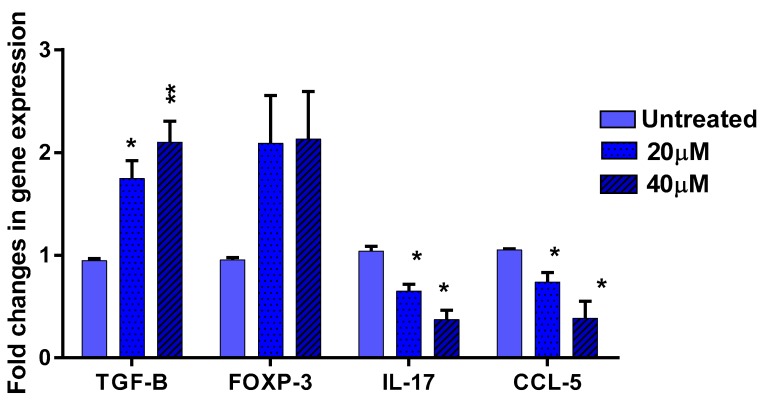
The effect of EGCG treatment was studied on a group of inflammatory-regulatory genes involved in IP in immortalized lymphocytes. Following induction with IFN-γ, Jurkat cells were treated with 40 mM EGCG for 48 h and expression of IL-17, CCL-5, FOXP3, and TGF-β investigated by Q-PCR. CT values were normalized to GAPDH and differential expression (2^−∆∆CT) of AA candidate genes in EGCG treated samples was calculated against untreated samples. Data represented as mean ± SEM (*n* = 4). Significant difference * *p* < 0.05, ** *p* < 0.01.

Alopecia areata is the consequence of an imbalance between the inflammatory and regulatory arms of the immune system, causing IP collapse. Therefore, the effect of EGCG on the expression of key inflammatory and regulatory candidate molecules involved in AA pathogenesis, such as IL-17, CCL-5, TGF-β, and FOXP-3, was investigated by Q-PCR in T cells. CCL-5 expression was significantly reduced (*p* < 0.05). This was not surprising as CCL5 is activated by pSTAT1 downstream in the IFN-γ pathway. The Th17 marker IL-17 was also significantly increased in both cell lines (*p* < 0.05). On the other hand, expression of the anti-inflammatory cytokine TGF-β was strongly enhanced (*p* < 0.01). The expression of T-reg marker FOXP3 was also increased but this did not reach statistical significance (Figure 6).

## 4. Discussion

IFN-γ is pivotal in inducing IP collapse in the hair follicle and inhibiting its signaling pathway is the target of many therapeutic options. JAK inhibitors including ruxolitinib, tofacitinib, and baricitinib were recently used in clinical trials of several inflammatory/autoimmune diseases, including skin diseases such as psoriasis [76] and AA [57]. JAK inhibitors are efficient but have relatively high cost and they can have a range of significant side-effects [66]. For instance, ruxolitinib is a broad JAK (1 and 2) inhibitor with the potential to modulate the signaling pathway of cytokines, including IL-6, IL-10, IL-22, and IL-3 [77]. Ruxolitinib side-effects include reactivation of tuberculosis, thrombocytopenia, anemia, and there is a risk of other unknown long-term side-effects [78]. We now propose EGCG as a potential candidate for AA treatment mainly based on its anti-inflammatory properties and encouraged by its good safety profile [74,79]. EGCG has an inhibitory effect on IFN-γ signaling via inhibiting STAT1 phosphorylation, which has been demonstrated by many studies [69,80,81]. We therefore sought to establish whether EGCG acts directly on STAT1 and whether STAT1 inhibition can restore IP in HF.

In this study, AA PMBCs produced significantly higher levels of IFN-γ compared to HC, in keeping with previous findings [82,83,84]. Involvement of STAT1 in IFN-γ signaling was previously suggested by the high levels of p-STAT1 detected in lymphocytes around the affected HF [57]. However, it was not known whether this was due to an increase of p-STAT1 in circulating lymphocytes or a phenomenon restricted to skin lymphocytes. In this study, we found that the increased level of p-STAT1 in HF of AA patients is likely to be the result of an increase of p-STAT1 in circulating lymphocytes.

As both JAK1 and JAK2 catalyze STAT1 phosphorylation at Tyr701 [85], we investigated the effect of EGCG on JAK1 and JAK2 expression and found that EGCG inhibits specifically JAK2 expression but not JAK1. EGCG specifically blocks JAK2 and therefore will potentially be safer and more efficient as the broad JAK1/JAK2 inhibitors block the IFN-γ pathway as well as other pathways including IL-2, IL-6, IL-10, IL-23, and erythropoietin (Epo) [86]. The specificity of EGCG as a pure JAK2 inhibitor does not reduce its efficiency as this was accompanied by reduction in STAT1 phosphorylation at Tyr701 in both cell lines, as shown by our data. p-STAT1 interacts with IRF-1, which is a key regulator in IFN-γ signaling, and IFN-γ induced MHC I expression has been shown to be mediated by IRF-1 [87,88]. Furthermore, the EGCG inhibitory effect on p-STAT1, leading to the significantly decreased IRF-1 expression, which we observed in HaCat cells, is in keeping with the marked reduction in its protein level, as also demonstrated by Watson and colleagues [81] in colonic epithelial cells.

It is well known that activation of IFN-γ/STAT1/IRF-1 signaling leads to the activation of HLA class I and class II genes, in particular *HLA-B* and *HLA-DRB1* [89]. Interestingly, we found that *HLA-B* expression was significantly reduced after treating HaCat cells with 40 μm EGCG. *HLA-B* expression is a major factor controlling immunological balance in tissues manifesting IP. For instance, low corneal HLA-B27 is an important contributor to ocular immune privilege as demonstrated in HLA-B27 transgenic mice with ocular inflammation [90]. We have previously shown in AA that there was an aberrant expression of HLA-DR in the pre-cortical matrix and dermal papilla (DP) of lesional anagen follicles [33,34]. It has also been shown that MHC class I expression is at a very low level in the proximal epithelium of healthy anagen HF [91] but becomes highly expressed in AA lesional tissue [38].

The reduction of p-STAT1 was accompanied by a significant decrease in Th1 cells as well as a non-significant decrease in Th17 cells. These findings are in keeping with two other recent studies [55,92], underlining the possible role of these T cell subsets in the disease pathogenesis. In the light of this increasing evidence supporting the role of CD8+ cells in AA, our novel finding that EGCG has a significant effect on reducing NKG2D+ cells could have a significant impact in understanding the mechanisms underlying the pathogenesis of AA. In fact, the NKG2D receptor, which is expressed only on the surface of activated CD8+ cells, has been previously implicated in AA by virtue of upregulation of its ligands ULBP3 and MICA in the dermal papilla and dermal sheath of AA HFs compared to normal [2,56]. NKG2D ligands activate the cytotoxic activity of CD8+ T cells [2,57]. Such activation is followed by IFN-γ production by cytotoxic CD8+ NKG2D+ T cells mediating inflammation and HF damage [57]. Our findings shed more light on the role of cellular immunity mediated by T-lymphocytes in the pathogenesis of AA and suggest good potential for EGCG as a possible therapeutic agent.

## 5. Conclusions

Our findings have demonstrated that 20–40 µm of EGCG added to cultured HaCat cells is capable of reducing the expression of pSTAT1 and downstream genes in the IFN-g pathway, including *IRF-1*, *HLA-DR*, and *HLA-B*. These results confirm the importance of the IFN-g pathway in the pathogenesis of AA, and inhibiting this signaling pathway could be an efficient therapeutic target. This has already been shown to be the case with JAK inhibitors such as ruxolitinib, tofacitinib, and baricitinib. However, JAK inhibitors are relatively expensive and could present a range of significant side-effects. Therefore, it would be interesting to run side-by-side assays with EGCG and JAK inhibitors in a more temporally dynamic system such as the hair follicle. This could be followed by clinical trials, which would validate the in vitro data. We also showed, in ex-vivo experiments that 20 µM added to PBMCs from AA patients significantly reduced CD4+ C119 positive (Th1) cells as well as CD8+ NKG2D+ subset cells. These findings reinforce the importance of the immunological aspect in the pathogenesis of AA and could represent a significant step towards the development of this molecule in targeting these specific subsets of lymphocytes.

## Supplementary Materials

The following are available online at http://www.mdpi.com/1660-4601/15/12/2882/s1, Figure S1: The mean percentage (%) of viable cells in HaCat and Jurkat cell lines after treatment with different concentrations of EGCG (10, 20, 40, 60 and 100 µM) for 48 h, Figure S2: Morphological features of HaCat cells treated with EGCG, Figure S3: Morphological features of Jurkat cells treated with EGCG, Table S1: Summary of cDNA synthesis protocol, Table S2: Primers used in the q-PCR reactions, Table S3: Thermal profile used in q-PCR reaction, Table S4: Alopecia areata patients’ details.
