# Supplementary material for: Epigallocatechin-3 Gallate Inhibits STAT-1/JAK2/IRF-1/HLA-DR/HLA-B and Reduces CD8 MKG2D Lymphocytes of Alopecia Areata Patients
Source: Int J Environ Res Public Health. 2018 Dec 15;15(12):2882. doi: 10.3390/ijerph15122882 (PMC6313664; doi:10.3390/ijerph15122882)
Supplement: Supplementary file 1 [file ijerph-15-02882-s001.pdf]

International Journal of
Environmental Research and Public Health

MDPI

Supplementary Materials:

Table S1. Summary of cDNA synthesis protocol.

Reaction	Temperature ◦C/duration	Aim
10 µl RNA+Primers+dNTPs	65 for 5mins	Denaturation
10 µl cDNA synthesis mix+ Random hexamer	50 for 50mins	cDNA synthesis
	85 for 5mins	Terminate reaction
1ml Rnase H	37 for 20mins	Remove RNA

Table S2. Primers used in the q-PCR reactions.

Gene	Forward sequence	Reverse Sequence
IL-17A	AGATTACTACAACCGATCCACCT	GGGGACAGAGTTCATGTGGTA
STAT1	GCAGGTTCACCAGCTTTATGA	TGAAGATTACGCTTGCTTTTCCT
IRF1	GCAGCTACACAGTTCCAGG	GTCCTCAGGTAATTTCCCTTCCT
IL10	TCAAGGCGCATGTGAACTCC	GATGTCAAACTCACTCATGGCT
FOXP3	CGGACCATCTTCTGGATGAG	TTGTCGGATGATGCCACAG
TGFB1	CTAATGGTGGAAACCCACAACG	TATCGCCAGGAATTGTTGCTG
HLA-DR	ATCATGACAAAGCGCTCCAACTAT	GATGCCCACCAGACCCACAG
HLA-B	CCGGACTCAGAATCTCCTCAG	AAACACAGGTCAGCATGGGAA
GAPDH	GGAGCGAGATCCCTCCAAAAT	GGCTGTTGTCATACTTCTCATGG
β-Actin	TCCCCCAACTTGAGATGTATGAAG	AACTGGTCTCAAGTCAGTGTACAGG
JAK-1	GCGGAGGGATCGACAAATGG	TGGGACATAGCTTAAAGAGGCA
JAK-2	CTCTTTGTCACAACCTCTTTGCC	TTGGAGCATACCAGAGCTTGG
CCL-5	CTCATTGCTACTGCCCTCTGCGCTCCTGC	GCTCATCTCCAAAGAGTTGATGTACTC

Table S3. Thermal profile used in q-PCR reaction.

Reaction	Duration/Temperature	Cycle
Hot start activation of polymerase	10 min at 95 °C	1
Denaturation	15 s at 95 °C	40
Annealing and elongation	1 min at 60 °C	
Dissociation curve	15 s at 95 °C	1
	15 s at 60 °C	1
	15 s at 95 °C	1

A 10µl final volume of real-time PCR reaction, containing 600nM of forward and reverse primers, was run in triplicate. The targeted sequence-specific amplification was detected using SYBER green detector and the thermal profile (Sup table 3). 5 µg total RNA was reverse transcribed to a first strand cDNA using SuperScript® III First-Strand Synthesis System kit using random hexamer (18080-051, Life Technologies) by following the manufacturer's protocol where cDNA synthesis mix contains 10X RTbuffer+ 20mM $MgCl_2$+ 0.1M DTT+ RnaseOUT+Glyceraldehyde-3-phosphate dehydrogenase (GAPDH) was run for each sample as an internal control to normalise any variation in RNA amount, and an NTC control was run to regulate any contamination. The PCR reaction was carried out in 384-well plates using the ABI Prism 7900HT Sequence Detection System (Applied Biosystems).

Table S4. Alopecia areata patients' details.

Code	Age	Age at onset AAO	Type of Alopecia
AA-S01	52	34	AU
AA-S02	37	25	AA
AA-S03	30	12	AA
AA-S04	41	21	AA
AA-S05	32	16	AA
AA-S06	62	28	AT
AA-S07	68	47	AA
AA-S08	51	45	AU
AA-S09	44	12	AT
AA-S10	51	30	AU
AA-S11	38	26	AA
AA-S12	54	21	AA
AA-S13	36	34	AU
AA-S14	36	34	AA
AA-S15	50	38	AU
AA-S16	50	47	AT
AA-S17	54	32	AA
AA-S18	63	32	AU
AA-S19	50	17	AT
AA-S20	51	21	AT

AA: alopecia areata; AAO: age at onset; AA: patchy AA, AT: alopecia totalis; AU: alopecia universalis.

Optimization of EGCG dosage

EGCG has been used topically at concentrations between 40–660 µM without inducing dermal toxicity (Zhao et al., 2015), with, an optimal dose previously used in cell culture of 50–75 µM with HaCat cells (Zhu et al., 2014) and 100 µM with epidermal kerantinocytes (Hsu et al., 2003). The first step in this study was to test the range of EGCG dosages that can be tolerated by the HaCat and Jurkat cell lines. 10, 20, 40, 60 and 100 µM EGCG concentrations were used to treat these cell lines for 24 and 48 h, with cell viability assayed by microscopic evaluation and by staining the dead cells with trypan blue to find the percentage of viable cells in each group.

Cell viability by Trypan blue

The effect of EGCG on cell viability was found to be dose-dependent, regardless of the duration of treatment, with data from 48 h treatment presented here, and data from 24 h treatment found in appendix 3. The viability of HaCat cells compared to untreated samples, reduced slightly when treating the cells with 10 µM EGCG and continued to drop gradually when increasing the dose of EGCG to 20, 40 and 60 µM. However, this reduction was mild and not statistically significant. A significant sudden drop in cell viability was observed in samples treated with 100µM EGCG ($p < 0.001$), where it declines to about 50% (Figure S1).

The same trend was seen in Jurkat cells, although a very significant toxic effect ($p < 0.001$) of EGCG was observed at a lower dose of EGCG (60 µM) for Jurkat cells, reducing to approximately 50% cell viability, which dropped further to reach about 30% when increasing the dose to 100 µM. Therefore, it was concluded that 10, 20 and 40 µM EGCG dosages do not show statistically significant adverse effects on cell viability in either cell line.

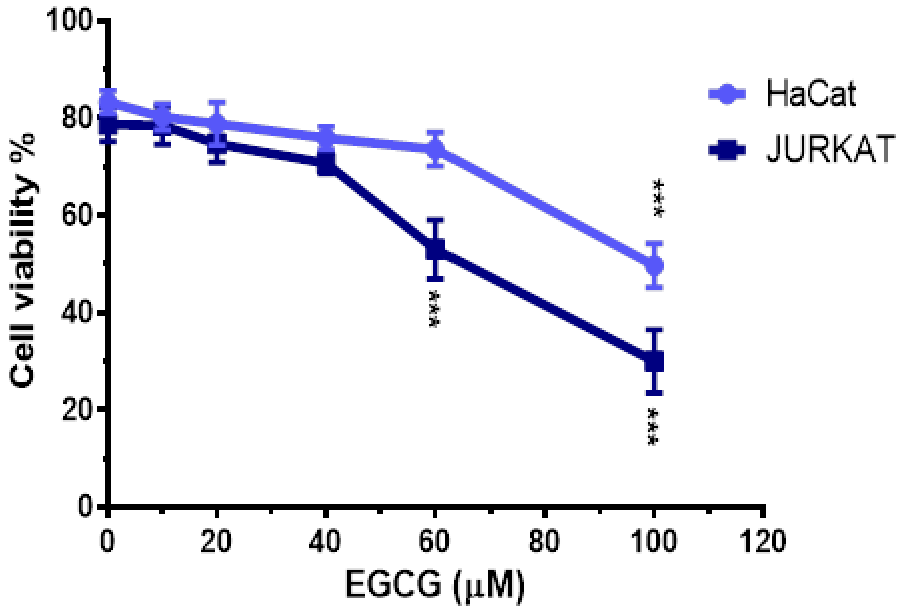

Figure S1. The mean percentage (%) of viable cells in HaCat and Jurkat cell lines after treatment with different concentrations of EGCG (10, 20, 40, 60 and 100 μM) for 48 h.

Slight reduction in viability can be seen in both cell lines after treatment with lower doses of EGCG 10, 20, 40 μM (not statistically significant) while a significant drop started to be seen at 100 μM in HaCat cells and 60 μM in Jurkat cells. The experiment was repeated three times and mean and SD were calculated. Asterisks denote a significant reduction in cell viability, *** $p < 0.001$.

Microscopic assessment of cell viability

To confirm the viability assay results, microscopic assessment of HaCat and Jurkat cells was performed after 48 h of treatment with EGCG. HaCat cells treated with 10, 20 and 40 μM EGCG displayed the same morphology as the untreated control group, where the cells proliferated in a compact monolayer in a relatively non-structured pattern. On the other hand, 60 μM and 100 μM EGCG treated cells showed marked disruption in the monolayer of cultured cells, and a reduction in cell density with adherent cells displaying a longer, stretched morphology (Figure S2). The Jurkat cells tend to be round and clump together to form grape-like colonies in untreated samples, and the same morphology can be seen for 10, 20 and 40 μM EGCG treated samples (Figure S3). As with the HaCat cells, Jurkat cells cannot tolerate the higher doses of EGCG (60 μM and 100 μM) and the cells appeared as discrete entities with small particles floating in the media, which are probably apoptotic bodies when compared to the microscopic images by Ivan et al. (Ivan et al., 2014). These results indicate the adverse effect of EGCG on cell viability, and thus its toxicity at higher dosages.

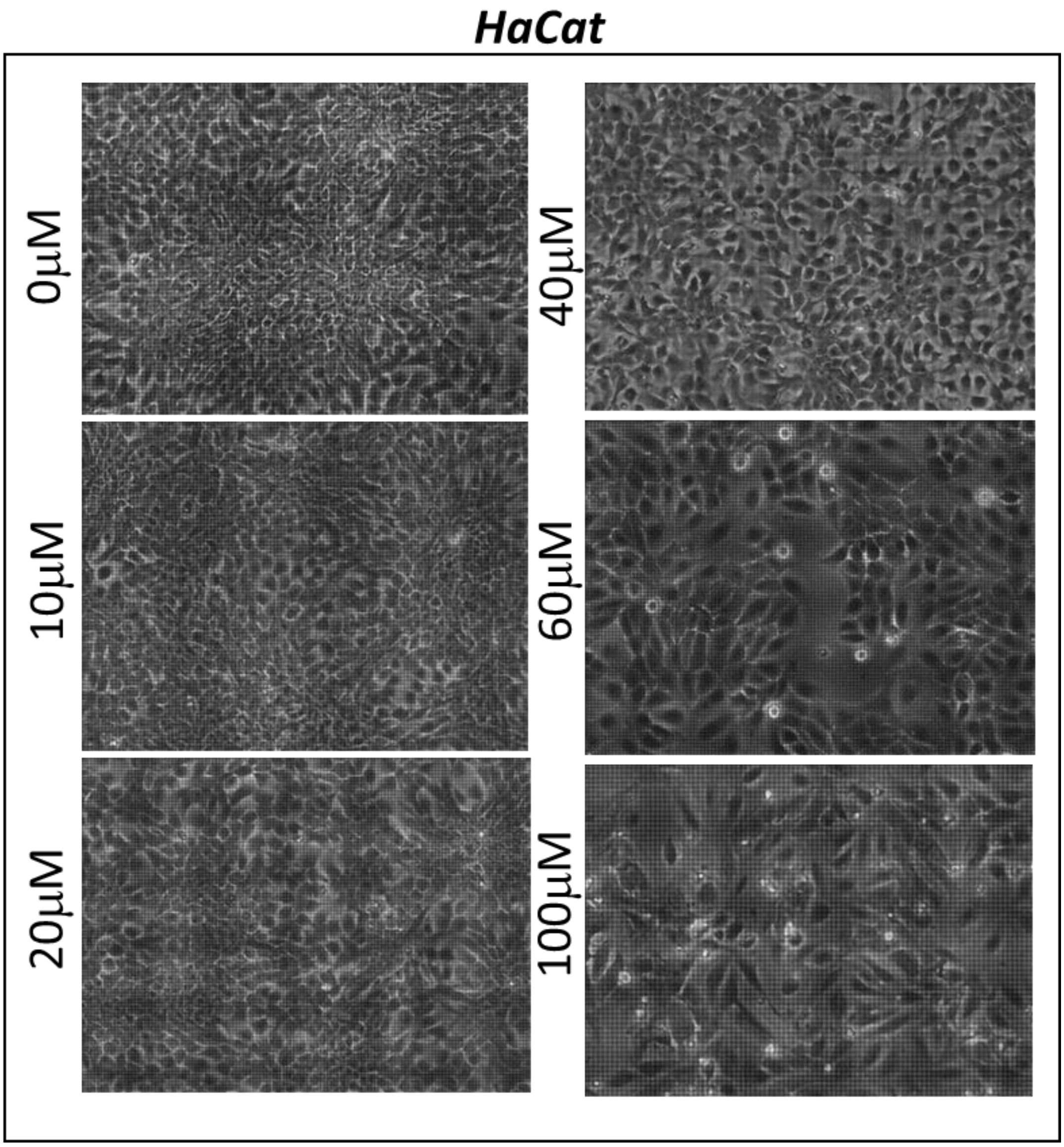

Figure S2. Morphological features of HaCat cells treated with EGCG**.**

Cells treated for 48 h with 10, 20, 40, 60 or 100 μM EGCG or left untreated as a conrol were examined under a light microscope at 10X magnification. The morphology in control versus 10, 20, 40 μM EGCG-treated cultures is relatively similar; however, 60 and 100 μM EGCG alters the colony morphology forming.

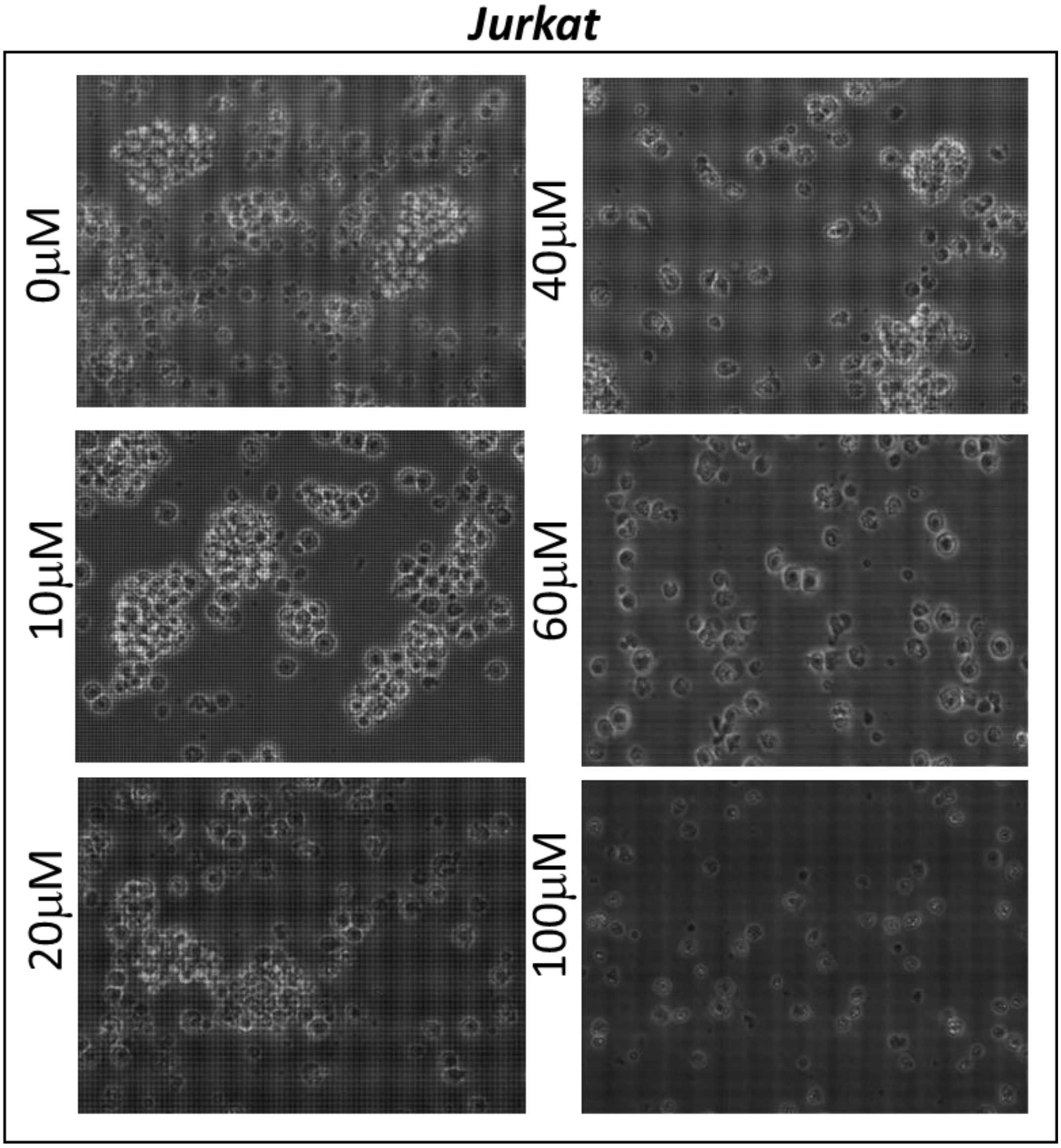

Figure S3. Morphological features of Jurkat cells treated with EGCG.

Cells treated for 48 h with 10, 20, 40, 60 or 100 µM EGCG or left untreated as a conrol were examined under a light microscope at 20X magnification. The morphology in control versus 10, 20 , 40 µM EGCG-treated cultures is relatively similar with grape-like colonies; however, 60 and 100 µM EGCG alters colony morphology forming less compact colonies with more discrete cells .

Based on the viability assay and microscopic findings, we choose 40 µM of EGCG as an optimal dose that can tolerated by HaCat and Jurkat cells without causing significant cell death. This dose was used in the subsequent experiments to investigate its effect on the expression of key molecules involved in JAK-STAT pathway.

1. ZHAO, H., ZHU, W., JIA, L., SUN, X., CHEN, G., ZHAO, X., LI, X., MENG, X., KONG, L., XING, L. & YU, J. 2015. Phase i study of topical epigallocatechin- 3- gallate (EGCG) in patients with breast cancer receiving adjuvant radiotherapy. *British Journal of Radiology*, 89, 1058.
2. ZHU, W., XU, J., GE, Y., CAO, H., GE, X., LUO, J., XUE, J., YANG, H., ZHANG, S. & CAO, J. 2014. Epigallocatechin- 3- gallate (EGCG) protects skin cells from ionizing radiation via heme oxygenase- 1 (HO- 1) overexpression. *Journal of Radiation Research*, 55, 1056-1065.
